# Conservation and novelty in the microRNA genomic landscape of hyperdiverse cichlid fishes

**DOI:** 10.1038/s41598-019-50124-0

**Published:** 2019-09-25

**Authors:** Peiwen Xiong, Ralf F. Schneider, C. Darrin Hulsey, Axel Meyer, Paolo Franchini

**Affiliations:** 1 Chair in Zoology and Evolutionary Biology, Department of Biology, University of Konstanz, 78457 Konstanz, Germany; 2 Present Address: Marine Ecology, Helmholtz-Zentrum für Ozeanforschung Kiel (GEOMAR), 24105 Kiel, Germany

**Keywords:** Evolution, Evolutionary genetics

## Abstract

MicroRNAs (miRNAs) play crucial roles in the post-transcriptional control of messenger RNA (mRNA). These miRNA-mRNA regulatory networks are present in nearly all organisms and contribute to development, phenotypic divergence, and speciation. To examine the miRNA landscape of cichlid fishes, one of the most species-rich families of vertebrates, we profiled the expression of both miRNA and mRNA in a diverse set of cichlid lineages. Among these, we found that conserved miRNAs differ from recently arisen miRNAs (i.e. lineage specific) in average expression levels, number of target sites, sequence variability, and physical clustering patterns in the genome. Furthermore, conserved miRNA target sites tend to be enriched at the 5′ end of protein-coding gene 3′ UTRs. Consistent with the presumed regulatory role of miRNAs, we detected more negative correlations between the expression of miRNA-mRNA functional pairs than in random pairings. Finally, we provide evidence that novel miRNA targets sites are enriched in genes involved in protein synthesis pathways. Our results show how conserved and evolutionarily novel miRNAs differ in their contribution to the genomic landscape and highlight their particular evolutionary roles in the adaptive diversification of cichlids.

## Introduction

Regulatory changes are major drivers of adaptive diversification^1^. The genomic mechanisms determining levels of gene expression are likely critical to adaptive phenotypic innovations, but we are only now beginning to uncover how different kinds of regulatory mechanisms contribute to diversification. For instance, in the past two decades, microRNAs (miRNAs), short ~22-nucleotide (nt) noncoding RNAs, have emerged as key post-transcriptional regulators of gene expression. By binding to targeted mRNAs, miRNAs can direct the downregulation of gene expression through mRNA cleavage and/or translational repression^2^. However, although much of the role of miRNA in adaptive divergence could stem from the relatively recent origin of novel miRNAs, our information about miRNAs for many groups is largely confined to miRNAs that are highly conserved and documented in comparative genomic databases. In general, it is unclear how the genomic organization of these highly conserved miRNAs differs from that of miRNAs that have arisen more recently, as well as how novel miRNAs might influence the phenotypic diversification of highly diverse groups of organisms, such as the extremely species-rich cichlid fishes.

The genes that encode miRNAs are endogenously transcribed into primary miRNAs (pri-miRNAs) and further cleaved into precursor miRNAs (pre-miRNAs) by the Drosha endonuclease. Pre-miRNAs contain ~70 nt and have typical stem-loop structures, or so-called hairpins^3^. Mature miRNAs are produced by the Dicer endonuclease from one or both arms of pre-miRNA hairpins, then loaded onto the Argonaute (ago) proteins to produce the effector RNA-induced silencing complex (RISC)^4^. In animals, miRNAs usually bind to the 3′ untranslated regions (3′ UTRs) of targeted mRNAs with Watson-Crick pairing to the “seed” regions, which are at 5′ regions of the miRNAs centered on 2–7 nt^5^.

Hundreds to thousands of miRNAs genes have been discovered in animals, plants, fungi, and even viruses, which suggests an evolutionarily ancient origin of miRNAs and their regulatory mechanisms^6–8^. Many miRNA gene families are conserved among species and this conservation serves as a widely used criterion to identify homologous miRNA genes^9–11^. Moreover, the target sites of miRNAs also show high conservation among closely related species, indicating that similar pathways and biological processes among evolutionarily divergent species are likely regulated through the same conserved miRNAs^8,12^. However, variants of miRNAs, termed isomiRs, can originate during processes such as RNA editing, miRNA transfer, or imprecise cleavage of Drosha or Dicer enzymes^13–15^. Additionally, novel miRNAs can evolve from the duplication of existing miRNA genes^16,17^, and arise from the incidental formation of hairpins in DNA molecules^18,19^. It has been reported that new miRNAs emerge frequently, but only a few of these genomic novelties are likely to be retained and/or expressed^20,21^. Despite the ancient origin and highly conserved mechanism of miRNAs, the understanding of how new miRNAs arise and take on novel functions among closely related species and how they might contribute to phenotypic diversification and novelties remains limited.

In animals, several miRNAs can often be found to be clustered on a single polycistronic transcript^22,23^ and the distance of two neighboring miRNAs within a cluster is often less than 5 kilobases (kb)^21^. One outcome of this proximity is that a cluster of miRNA genes can be controlled by the same transcription factors (TFs)^24^ and therefore may be regulated by the same or similar regulatory pathways^25^. It has also been hypothesized that miRNAs in the same cluster have associated regulatory functions and target the same or related genes^26–28^. Because mechanisms such as tandem duplication might be one of the processes leading to increased numbers of similar sequences in clusters, one might expect that more conserved miRNAs should show greater degrees of physical clustering in the genome as compared to more recently evolved miRNAs.

Most protein-coding genes are regulated by miRNAs. For instance, more than 60% of human genes contain targets of conserved miRNAs^12^. Increasing evidence underscores the important roles of miRNAs in many core cellular processes such as cellular differentiation, cell fate determination, proliferation and tissue differentiation^29–32^, as well as the wide involvements of miRNAs in diseases and cancers^33–36^. Serving as fundamental regulators in post-transcriptional gene expression, miRNAs are also thought to contribute to gene regulation during environmental adaptation, phenotypic diversification, and speciation^37–43^. One might speculate that, similar to protein coding loci, the number and diversity of miRNAs are strongly conserved across closely related taxa. Alternatively, novel miRNAs could be gained and lost relatively rapidly across closely related groups, especially when these groups are known to undergo extensive adaptive diversification as is found in cichlid fishes. Thus, miRNAs could be key drivers of diversification in rapidly evolving lineages such as cichlid fishes.

With their astonishing level of phenotypic diversification and extremely high rates of speciation, cichlid fishes are one of the best models of vertebrates to study rapid evolution^44,45^. The most diverse radiations of cichlids are found in Africa and in Central and South America. Approximately 1,500 known cichlid species live in the African great lakes, while over 600 cichlids occur in Central and South America^46–48^. There is increasing evidence to suggest that gene regulation might drive much of the evolution of phenotypic divergence in cichlids^46,49–51^. In our previous study on the evolution of miRNA target sites in the Midas cichlid species complex, we suggested that different selective pressures might drive diversification among lineages of these cichlids^52^. Moreover, in another study, when we compared cichlids with other teleost fishes, we discovered that a greater number of regulatory interactions at the miRNA level could be found in cichlids^53^.

To further investigate the potential roles of miRNAs and their evolutionary consequences for the radiation of cichlids, we applied RNA-Seq techniques for both miRNA and mRNA. We characterized the sequences and features of miRNAs across species within the two largest lineages of cichlid fishes, those from Africa and those from the New World. We used extensive transcriptomic sequencing and bioinformatic screening to identify a large number of conserved and novel miRNAs. We predicted miRNA target sites and tested the correlated expression between miRNAs and their predicted target mRNAs. Furthermore, we examined in detail whether conserved miRNAs showed different expression levels on average compared to species-specific miRNAs in particular cichlids, and whether miRNA conservation is associated with the number of target sites as well as miRNA sequence variability. Also, we evaluated whether miRNA conservation is associated with miRNA clustering in the genome. Additionally, we assessed the general spatial distribution of miRNA target sites in cichlid 3′ UTRs, and again linked these distributions to miRNA conservation. Finally, we determined if there were any GO terms that were enriched for genes that accumulated the greatest number of novel miRNA target sites, which might indicate a particularly important role of those categories for the rapid diversification of cichlid fishes.

## Results

### RNA sequencing

Total RNA from 1 day post-hatch (1 dph) embryos was extracted from five African cichlids (*Maylandia zebra*, *Pundamilia nyererei*, *Astatoreochromis alluaudi*, *Astatotilapia burtoni, Neolamprologus brichardi*) and from three Neotropical cichlids (*Amphilophus zaliosus, Archocentrus centrarchus, Amatitlania siquia*). RNA-Seq and small RNA-Seq techniques were applied to six individuals of each species and used to produce the transcriptomic data for identifying transcripts, isolating miRNAs, and quantifying expression. For each species, we acquired between 28 M to 33 M paired-end reads of RNA-Seq data and between 30 M to 59 M single-end reads of small RNA-Seq data (Supplementary Table S1).

### Phylogenetic reconstruction

**Figure 1:**
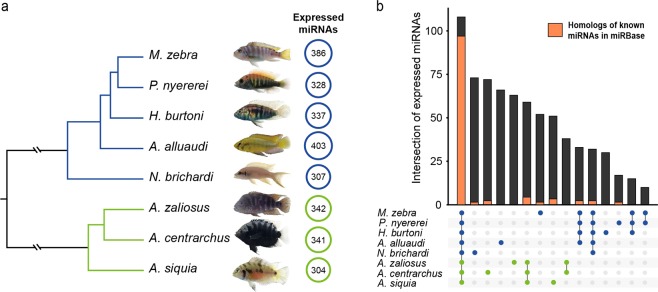
Identified miRNAs in eight cichlid species. (**a)** Phylogenetic tree of the focal cichlid fishes constructed using 2,262 one-to-one orthologous transcripts (all branches are 100% supported by bootstrap value). The circles indicate the number of expressed mature miRNAs detected in each species (circle size scaled accordingly). **(b)** Overview of miRNA numbers uniquely expressed in one species or shared among different monophyletic groups within the eight cichlids studied. Dots depict which lineages share the bar of miRNAs. Lines connect the dots of monophyletic groups that share particular miRNAs. The orange portion of each bar indicates the number of expressed miRNAs of our dataset that are homologous to known miRNAs in miRBase. In both figures, blue and green are used to highlight African and Neotropical cichlid species, respectively.

Based on sequence similarity, 2,262 one-to-one orthologous transcripts were identified in all focal species (Supplementary Table S2). For the comparative analyses, a species tree was inferred based on the aligned codon regions (1,057,619 bp) of these orthologous genes using maximum likelihood phylogeny reconstruction implemented in RAxML. The relative divergence times among species was estimated based on 393,366 four-fold degenerate sites and represented as the branch lengths in the phylogeny (Fig. 1a). The resulting topology was consistent with previously hypothesized relationships among these species^46,54–56^.

### Identification of miRNAs

Small RNA-Seq reads were used to identify miRNA genes from corresponding reference cichlid genomes^46^. After removing adaptors, the length distributions of small RNA-Seq reads had clear peaks at 22 nt (Supplementary Fig. S1), as expected if most of the reads were mature miRNAs. In the eight focal cichlid species, we identified a total of 2,748 expressed mature miRNAs (from 304 in *A. siquia* to 403 in *A. alluaudi*, Fig. 1a). Of these, 2,378 miRNA genes were identified in the six species with reference genomes (ranging from 363 in *N. brichardi* to 467 in *M. zebra*, Supplementary Table S3). Using their phylogenetically closest relatives’ genomes, we then identified 304 mature miRNA in *A. siquia* and 403 mature miRNA in *A. alluaudi*. A total of 108 mature miRNA genes were found to be “conserved”, i.e. shared by all eight cichlid species, but also a great number of miRNAs were identified as being “species-specific” (Fig. 1b and Supplementary Table S4). By comparing the expressed miRNA in our dataset to mature miRNA sequences from miRBase using similarity searches (see Methods for details), we found a large number of “novel” miRNAs (only 1,010 out of our 2,748 miRNAs were inferred to be homologous to miRNAs in miRBase, hereafter referred to as “known” miRNAs, Supplementary Table S3). Notably, most of these known miRNAs fall in the conserved category (90% of the conserved miRNAs, 97 out of 108, are present in miRbase), while a very small fraction of known miRNAs were non-conserved, that meant only present in some of the cichlids species (Fig. 1b). The parsimony analysis of miRNA gain and loss events also suggested that a large number of miRNAs were gained on lineage-specific branches (Supplementary Fig. S2).

### Expression patterns of miRNAs and mRNAs

**Figure 2:**
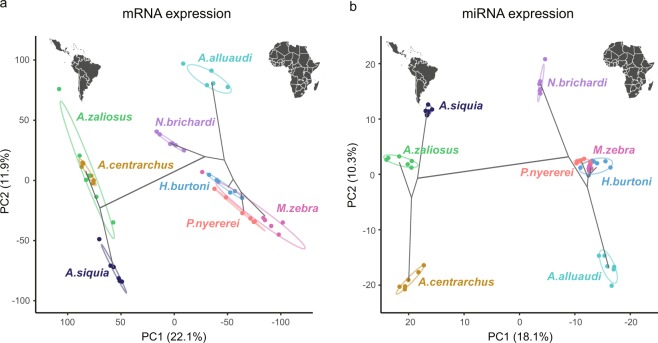
Principal component analysis (PCA) plot of RNA expression. In both **(a)** mRNA and **(b)** miRNA, PC1 explained around 20% of the total variation and PC2 explained approximately 10%. PC1 closely reflects the earliest phylogenetic split among these eight cichlid fishes (as depicted with the black lines) and separated cichlid groups from the Neotropics (on the left) and Africa (to the right) into distinct lineages.

The expression of miRNAs and mRNAs was quantified and subsequently normalized for each individual. Then principal component analyses (PCA) were performed on the normalized expression of miRNAs and mRNAs (Fig. 2). The first principal component explained 18.1% and 22.1% of the total variances of the miRNA and mRNA expression, respectively. In both PCAs, the divergence in the first two principle components generally reflected the phylogenetic history among the focal cichlids with a particularly distinct split between the African and the Neotropical cichlids along PC1.

### Differentiation of miRNA features

**Figure 3:**
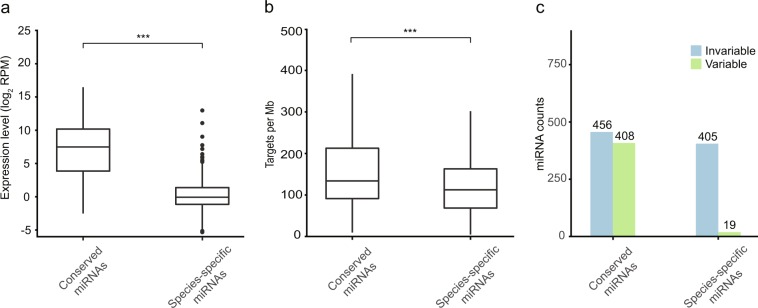
Differentiation in characteristics of conserved and species-specific miRNAs. miRNAs in cichlid fishes were grouped by their level of evolutionary conservation: (1) miRNAs present in all species: “conserved” miRNAs; (2) miRNAs present in single species: “species-specific” miRNAs. **(a)** The relationship between miRNAs conservation and their expression values shows that miRNAs conserved in all species tend to have higher expression levels. **(b)** The miRNAs conserved across all species also tended to have a greater number of target sites than species-specific miRNAs. The pairwise Mann–Whitney–U significance levels of comparisons is shown (*p < 0.05; **p < 0.01; ***p < 0.001). **(c)** The relationship between miRNAs conservation and their variability: miRNAs are defined as variable miRNAs if multiple isomiRs are detected in the focal cichlids. The same analyses were performed by including a third group, miRNAs present in at least two species but not in all eight species (Fig. S2).

After categorizing the miRNAs as either conserved or non-conserved as detailed above, we quantified the expression, the number of target sites, and the variability of mature sequences and compared these different groups of miRNAs. The conserved miRNAs had the highest average expression levels, while the species-specific miRNAs showed the lowest expression (pair-wise Mann–Whitney–Wilcoxon tests: all p < 0.001; Fig. 3a, Supplementary Fig. S3). A similar trend was observed in miRNA target sites (Fig. 3b, Supplementary Fig. S3), providing evidence for the hypothesis that highly conserved miRNAs have more targets than other groups of miRNAs (pair-wise Mann–Whitney–Wilcoxon tests: all p < 0.001). Variants of particular miRNAs were also observed, both inter- and intra-species. Variants were more frequently recognized in the group of miRNAs present in all species (51 out of 108 miRNAs; 47.2%) than in species-specific miRNAs (19 out of 424; 4.5%; χ^2^ = 133, df = 1, p < 0.001; Fig. 3c, Supplementary Fig. S3).

### Clustering of miRNA genes

**Figure 4:**
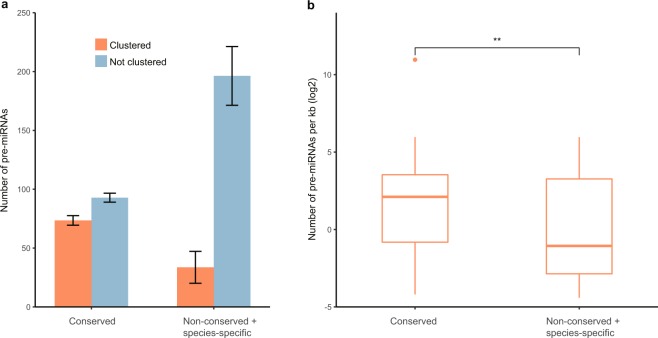
Levels of clustering of miRNA genes. **(a)** miRNA genes (pre-miRNA) in different levels of conservation show differences in clustering patterns. The conserved miRNA genes (those shared by all the eight cichlid species) occur more frequently in clusters than non-conserved miRNA genes do (chi-square test, p < 0.001). **(b)** The density of conserved miRNA gene clusters is significantly higher than the density of non-conserved clusters (t-test, p = 0.005).

The clustering pattern of miRNA genes were investigated in the six cichlids with a complete genome assembly (*M. zebra, P. nyererei, H. burtoni, N. brichardi, A. zaliosus*, and *A. centrarchus*). In these genomes, physical distance was calculated for every pair of pre-miRNAs on the same strand of the same scaffold and pre-miRNAs were categorized as clustered together if the distance between two precursor miRNAs was shorter than 5 kb. As only a very few species-specific pre-miRNAs clustered together, we compared the clustering patterns of conserved and non-conserved pre-miRNAs. It was observed that 165 (44.19%) conserved pre-miRNAs were clustered, but only 202 (14.64%) non-conserved pre-miRNAs were clustered (Fig. 4a). Most of the conserved and non-conserved pre-miRNAs clustered within each category (i.e. conserved and non-conserved miRNAs were present in different clusters). The density of conserved miRNA clusters (on average 38.8 pre-miRNAs per Kb) was significantly higher than that for novel miRNA clusters (on average 10.7 pre-miRNAs per Kb) (Fig. 4b, t-test: p = 0.005).

### Spatial distribution of miRNA targets

**Figure 5:**
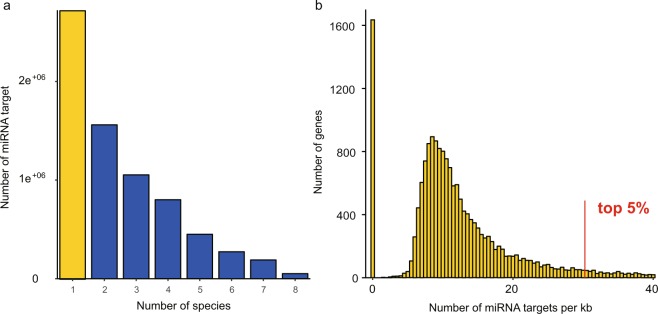
Evolutionary patterns of miRNA target sites. (**a)** The number of predicted miRNA target sites. Species-specific target sites are shown in yellow bar and other target sites with different level of conservation are shown in blue bars. **(b)** Distribution of species-specific miRNA targets in genes. For each gene, the density of species-specific targets was counted based on the length of the 3′ UTR. Some genes at the right tail exhibit extremely high emergence rates of species-specific targets. The top 5% genes were selected for GO enrichment test.

**Figure 6:**
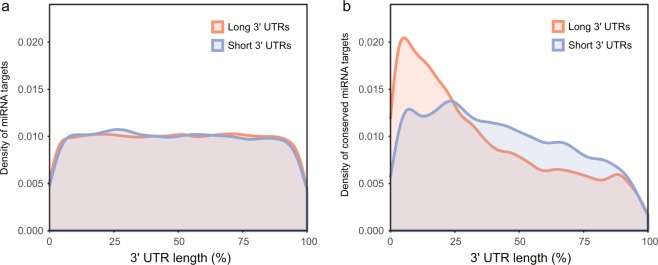
Distribution of miRNA target sites identified on the 3′ UTRs. (**a)** The density (width = 0.1) of all miRNA genes identified in the eight cichlid species. Target sites as a whole are generally evenly distributed across 3′ UTR sequences. **(b)** The density (width = 0.1) of the conserved miRNA targets (those shared by the eight cichlid species). The distribution of miRNA target sites that are conserved in all eight cichlid species is skewed towards being more frequently located near the beginning of the 3′ UTR. In both graphs, the x-axis represents the relative position of the 3′ UTRs, from stop codon on the left to poly-A tail on the right. The “long 3′ UTRs” are the set of 3′ UTRs longer than the median (shown in red), while the “short 3′ UTRs” are the ones shorter than the median (shown in blue).

The 3′ UTRs of 18,233 orthologous transcripts were aligned and approximately 18 million target sites of 2,457 mature miRNAs were predicted using TargetScan6 (Fig. 5a). To investigate the distribution of target sites, the relative position of each target site was calculated and the distribution of miRNA target sites was estimated using the *density* function in R with default bandwidth. Then a linear regression on the density from 10% to 90% positions of the 3′ UTRs was performed and the slope was estimated. Target sites of miRNAs were generally evenly distributed (with very low absolute slopes, Supplementary Table S5) along the whole length of 3′ UTRs (Fig. 6a). However, when we focused on the target sites that are conserved in all investigated cichlids, we found that these were disproportionately more frequent in the 5′ end of 3′ UTR and therefore were more often located closer to the coding regions of their target genes (Fig. 6b, Supplementary Table S5).

### Expressional correlation between miRNA-mRNA pairs

To estimate the association between miRNA and mRNA expression, we assessed the correlation between miRNAs and their target mRNA's expression. The Pearson’s correlation coefficients were calculated between the miRNA and mRNA expression of each individual if the targets were predicted in all focal cichlid species. As predicted from the hypothesis that miRNAs should often down-regulate mRNA expression, negative correlations between miRNAs and mRNAs were more often found than positive ones (Supplementary Fig. S4; chi-square test, p = 0.069). Further, the expression correlation of these miRNA-mRNA pairs was inferred within a phylogenetic framework, and a similar distribution of coefficients was found with more negative correlations (Supplementary Fig. S4; chi-square test, p = 0.066).

### The pattern of miRNA targets in cichlids

We finally tested how miRNA targets might be associated with the diversification patterns of cichlids. First, to examine the conserved target sites, 108 miRNAs present in all focal species and 2,262 one-to-one orthologous mRNAs were selected and a PCA was performed based on the target prediction of these miRNAs and mRNAs (Supplementary Fig. S5). The first PCAxis explained 40.6% of the variance, which mainly reflected the phylogenetic difference among the African and the Neotropical cichlids. Second, to examine the novel targets, we counted the emergence of species-specific miRNA target sites for each gene and normalized it by the length of the 3′ UTR. A large proportion of genes did not contain species-specific miRNA target sites, while the rest showed a nearly normal distribution skewed to the right tail (Fig. 5b). We, therefore, selected the top 5% genes on this right tail, which had the highest density of species-specific miRNA target sites and performed GO enrichment analysis on this subset of genes. By comparing these to the Nile tilapia dataset from Ensembl, GO terms were identified to be significantly biased towards peptide biosynthesis, amid metabolic process and translation (Table 1).

**Table 1:** Significantly enriched GO terms among the top 5% genes with highest emergence rate of species-specific miRNA targets.

p-value	term ID	GO type	name
0.00206	GO:0043603	Biological Process	cellular amide metabolic process
0.00569	GO:0043604	Biological Process	amide biosynthetic process
0.00315	GO:0006518	Biological Process	peptide metabolic process
0.00343	GO:0043043	Biological Process	peptide biosynthetic process
0.00306	GO:0006412	Biological Process	translation
0.0362	GO:0008150	Biological Process	biological process

These genes were aligned to the Ensembl release 92 Nile tilapia (Orenil1.0) protein dataset.

## Discussion

A substantial proportion of miRNAs are evolutionarily highly conserved in cichlids, but an even greater percentage of the “miRNAome” we identified here are not found in all cichlids, but rather are lineage-specific^6^. This finding suggests that phylogenetically imbalanced datasets that were originally used to establish the extent of miRNA conservation might have generally limited comparative studies on miRNAs across species^57^. This limitation might be particularly acute for teleost fishes, on which only a few studies so far have focused^46,52,58^. This study helps to fill this gap by providing a more thorough genome-wide atlas of miRNAs from both African and Neotropical cichlid fishes.

Across the phylogenetic diversity of cichlid species, we identified that the genomes of this group contain approximately 400 miRNAs per species (Fig. 1a, Supplementary Table S3). Brawand *et al*.^46^ reported approximately 270 miRNAs per species in the five African cichlids, which showed a high level of conservation when compared to other fishes^46^. Our results greatly expands the number of described miRNA sequences in cichlids. Through comparisons to the miRNA database, we also identified a number of miRNAs in cichlids that are already known in other species. However, we determined that around two out of three miRNAs identified here had not been described in either cichlids or other vertebrates previously. About 98% of these newly discovered miRNAs are also non-conserved in all cichlids (Fig. 1b).

The birth rate of miRNA genes might commonly be high, but only functionally important miRNAs can be selectively retained in the genome over long periods^20^. In our study, a total of 108 miRNAs were found to be present in all eight cichlids that we investigated and thus presumably arose prior to the split of African and Neotropical lineages. Such highly conserved and phylogenetically widespread miRNAs might be predicted to be under purifying selection over longer periods and might be expected to have conserved and essential functions. On the other hand, novel miRNAs tended to be expressed at lower levels and therefore might have more specific, although still potentially important, biological functions^59^. In cichlids, we observed both a significant decrease in expression levels when we compared conserved miRNAs with novel miRNAs as well as a gradual decrease in the number of targets from conserved miRNAs to novel miRNAs. Still, some outliers in the group of species-specific miRNAs had relatively high numbers of target sites. It has been reported that new targets are relatively easily gained and are widespread^5,60,61^. Our data suggest that acquiring new targets might be generally the first step for novel miRNAs to obtain biological functions. Future investigations of these outlier miRNAs that are novel, but show higher expression levels or a high number of target sites, could provide valuable insights into cichlid miRNA novelty and the function of these novel regulatory elements during diversification.

Although the sequences of miRNAs are generally highly conserved, variants of mature miRNAs found within or across species can be generated either by mutations on the miRNA genes or by modifications after transcription^62^. These variants of miRNAs (isomiRs) can have different targets and different influences on gene expression^63–65^. Nearly half of the miRNAs conserved in cichlids are variable, which likely allows for more regulatory possibilities. By contrast, only a few species-specific miRNAs are variable, which is probably due to the limited evolutionary time that these novel miRNAs have had to diversify. The conserved and non-conserved miRNA genes in cichlids display distinct clustering patterns, as these miRNA genes mainly cluster with the ones in the same category. The differences in how they physically co-localize in the genome suggest independent origins of conserved miRNAs and non-conserved miRNAs. The shorter physical distance between conserved miRNA genes may facilitate their expressional control and transcription.

Precise control of gene expression is fundamental for development and morphogenesis and in many cases likely to be rather complex because most genes are influenced by multiple regulators^66,67^. For instance, miRNAs not only target multiple genes, but also individual genes can be regulated by multiple miRNAs when respective binding sites are present^5,68,69^. Previous analyses have revealed distinct expressional patterns of conserved and non-conserved target sites: conserved miRNAs are co-expressed with their conserved target genes in the same tissue, while mRNAs with non-conserved target sites and the cognate miRNAs are often not expressed together^60,70,71^. These “targeting avoidance” mechanisms of non-conserved target sites are thought to be a major force driving the evolution of UTRs^60,70,72^. Here, we investigated the spatial distribution of conserved and non-conserved miRNA target sites on 3′ UTRs in cichlids. Despite there being no clear overall bias in the distribution of all miRNA target sites on 3′ UTRs, we observed that conserved miRNA target sites are more common in proximity to the 5′ end of the 3′ UTRs, particularly in longer 3′ UTRs (Fig. 6). The mechanisms generating this biased distribution of conserved miRNA target sites are unclear. However, one possible reason for this pattern might be that as the 5′ end of 3′ UTRs is closer to the coding region, purifying selection on coding regions may generally act against mutations in this region. As the increase of morphological complexity among organisms is thought to be associated with the elongation of 3′ UTRs^73^, our results on the distribution of conserved versus novel miRNA targets provide some insights into how 3′ UTRs might evolve: the 5′ end of 3′ UTRs is more ancient and evolutionarily conserved, while most of the new modifications, including lengthening of 3′ UTRs and gaining of novel miRNA target sites, occurrs predominantly at the 3′ end of 3′ UTRs.

We explored the expression correlation profiles for conserved miRNA-mRNA target pairs in cichlids. Despite finding both positive and negative correlations that were significant, we recovered more negative correlations between the expression of miRNA and the targeted mRNA (Supplementary Fig. S4). The global association between miRNA and mRNA expression can also differ in different species and tissues, showing the general complexity of these regulatory interactions^74–76^. Positive correlations between miRNA-mRNA pairs can be rationalized by miRNA-mRNA interaction mechanisms like co-transcriptional modules^77,78^ and upstream factors acting in feed forward loops that regulate miRNA and its target mRNA in the same direction^79–82^. In this study, we sequenced RNAs from 1 day post-hatch embryos because this stage, in which the majority of tissues are present and in active development, allowed us to capture the whole-organism transcriptional profile. However, this could represent an experimental limitation because the expression of miRNAs is known to be highly variable across tissues and might be concentrated to particular times during ontogeny^83,84^. The addition of more phylogenetic diversity in comparative studies of miRNA/mRNA co-expression and more transcriptional profiling at different ontogenetic stages could provide greater power to validate the empirical reality of bioinformatically inferred regulatory interactions.

Our PCA analysis of the target pattern of 108 conserved miRNAs and 2,262 one-to-one orthologous genes present in all species (Supplementary Fig. S5) emphasized the diversification of the miRNA target landscape between African and Neotropical cichlids. Cichlids of these two lineages cluster together along the main axis of variation, but both *A. siquia* and *A. alluaudi* are quite distinct from the other species along PC2, which is likely an artifact of the missing reference genomes for these two species. To further examine how the origins of novel target sites potentially contribute to the diversification of cichlids, we estimated the density of species-specific targets for each gene. Gene function analysis of the top 5% genes with the highest density of species-specific target sites showed significant enrichment in several interrelated biological processes of peptide synthesis. In our previous research, we showed that the 3′ UTRs of some ribosomal genes were particularly long and rapidly-evolving in cichlids compared to non-cichlid fishes, and proposed that more regulatory possibilities on 3′ UTRs of ribosomal genes made them important meta-regulators for cichlid diversification^53^. Here, by focusing on cichlid radiations only, we provided evidence from a different angle: protein synthesis related genes could be key drivers of lineage-specific diversification.

The evolution of conserved and novel miRNAs likely characterizes many lineages^85,86^. Understanding the diversity of the regulatory interactions between miRNA and mRNA will increasingly demand robust genomic assessments of the number of miRNAs, their targets, and comparative levels of expression. Additionally, greater efforts at characterizing the unique features of the genomic landscape of highly conserved miRNAs when compared to less conserved miRNAs will allow us to better understand how new miRNAs arise and how they could ultimately become conserved components of the genomic landscape. Novelty in genomic regulatory mechanisms could play an outsized role in rapidly diversifying groups like cichlid fishes.

## Conclusions

Using high throughput sequencing and large-scale genome mapping, we provided a genome-wide annotation of the miRNAomes of eight species from the two main lineages of cichlid fishes. We identified 2,378 miRNA genes and 2,748 mature miRNAs in these cichlids. Genome-wide comparisons among miRNAs demonstrated differences exist in expression, target numbers, sequence variability, and distribution of targets of conserved and non-conserved miRNAs. We uncovered expression associations between miRNAs and mRNAs globally. We also discovered the fast evolution of novel miRNA targets tend to be preferentially associated with the protein synthesis pathway and this could provide a genomic substrate for cichlid diversification. Our results provide new insights into the miRNA landscape of cichlid fishes and further our understandings on the roles of miRNA in diversification.

## Methods

### Samples collection

For this study, we used individuals raised in the laboratory from eight cichlid fish species for both RNA sequencing (RNA-Seq) and microRNA sequencing (miRNA-Seq): *Maylandia zebra* (Lake Malawi), *Pundamilia nyererei* (Lake Victoria), *Astatoreochromis alluaudi* (Lake Victoria and surrounding rivers), *Astatotilapia burtoni* and *Neolamprologus brichardi* (Lake Tanganyika) from East Africa; *Amphilophus zaliosus*, *Archocentrus centrarchus* and *Amatitlania siquia* from the Neotropics. Broods from the eight species were produced in the University of Konstanz animal facility (TFA) and sampled at 1 day post-hatch (1 dph). In total, 48 samples (six embryos per species) were manually de-yoked using sharp needles and processed for downstream RNA extraction and library preparation. All animal samples used in this study were collected in accordance with local regulations and guidelines (Tierforschungsanlage Konstanz, Anzeige T16-13).

### RNA extraction, library preparation, RNA sequencing

Total RNA from each sample was isolated using a Qiagen RNeasy Mini Kit (Qiagen, Valencia, USA) and small non-coding RNA molecules were retained by using 100% ethanol in the final washing steps. A FastPrep-24 homogenizer (MP Biomedicals) was used to process approximately 50 µg of each sample (30 sec at 4.0 m/s). A Bioanalyzer 2100 (Agilent Technologies, Palo Alto, USA) was used to check RNA quality, while its quantification was carried out with a Qubit v2.0 fluorometer (Life Technologies, Darmstadt, Germany). High-quality RNA samples (RIN value > 8.0) were used to construct RNA-Seq and miRNA-Seq sequencing libraries. For RNA-Seq, the Illumina TruSeq RNA sample preparation kit v2 (Illumina, San Diego, USA) was used to process 200 ng of total RNA in order to construct 48 sequencing libraries. Bar-coded libraries were paired-end sequenced (2 × 150 bp) in two lanes of an Illumina HiSeq2500 platform at the Tufts University genomic facility (TUCF Genomics, Boston, USA). For miRNA-Seq, the NEBNext Small RNA Library Prep Set (New England Biolabs, Beverly, USA) was used to construct 48 barcoded miRNA libraries from the same total RNA used for the RNA-Seq approach. Single-end sequencing (50 bp) was carried out in an Illumina HiSeq2500 at TUCF Genomics.

### Genomic resources

The genome assemblies of the African species *Maylandia zebra* (GCA_000238955.5), *Pundamilia nyererei* (GCA_000239375.1), *Astatotilapia burtoni* (also known as *Haplochromis burtoni*, GCA_000239415.1) and *Neolamprologus brichardi* (GCA_ 000239395.1) were retrieved from NCBI, while the genome, the transcript and the protein datasets of the African Nile tilapia *Oreochromis niloticus* (GCA_000188235.1) were downloaded from Ensembl release 92. Also, we used the unpublished genome assemblies of the Neotropical species *Amphilophus citrinellu*s and *Archocentrus centrarchus* for mapping the Neotropical cichlids’ RNA (Xiong and Franchini *et al. In preparation*).

### Identification of putative miRNAs

Raw reads from small RNA sequencing were processed to trim adaptors using the FASTX-Toolkit v0.0.14^87^. For each species, the reads from each individual were merged to identify putative species-specific miRNAs using miRDeep2.0.0.8^88^. Specifically, the reads were mapped to the corresponding genome using the miRDeep2 *mapper.pl* module with default parameters. As reference genomes are not yet available for *A. alluaudi*, *A. siquia* and *A. zaliosus*, we used other closely related species as references: (1) the genomes of *M. zebra*, *P. nyererei*, and *A. burtoni* were used for *A. alluaudi*; (2) the genomes of *A. citrinellus* and *A. centrarchus* were used for *A. siquia*; (3) the genome of *A. citrinellus* was used for *A. zaliosus*. For *A. alluaudi* and *A. siquia*, we combined the results obtained from the different genomes and kept the non-redundant sets of miRNAs. Mature miRNAs of fish were downloaded from miRBase v21^6^ and used as known mature miRNA set in the miRDeep2 analysis. The miRNAs with the miRDeep2 score (a value that represents the log-odds probability of a sequence being a genuine miRNA precursor versus the probability that it is a background hairpin) greater than or equal to 10 in any species were retained.

The mature miRNAs were defined into miRNA families by performing all-against-all comparisons using the *ssearch36* module in the FASTA package^89^ with e-value cutoffs of 0.01. The seed region of miRNA is crucial for target recognition. Thus, if mature miRNAs in the same miRNA family shared identical 2–8 nt seed sequence at the 5′-end, then they were considered as same miRNA gene iso-miRs^90–93^. Using the same approach, the precursor miRNAs were assigned into miRNA gene families using all-against-all *ssearch36* with e-value cutoffs of 0.005 (different e-values were used due to the different sequence length).

### Annotation, classification, and clustering of miRNAs

To annotate the putative cichlid miRNAs, we compared the sequences of mature miRNAs using *ssearch36* (-E 0.01 -C 25 -3) to the mature miRNAs downloaded from miRBase v21^6^ respectively. Using this framework, we defined a miRNA as “known” depending on the presence of its homologous sequence in miRBase, and as “novel” when it was only found in our dataset. We are aware that “novel” does not necessarily mean that a miRNA is novel to cichlids, as these miRNAs could be present in other species that are not yet represented in miRBase and/or in the species represented in miRBase, but have not yet been discovered so far. The inference of miRNA gain and loss events across the phylogeny was performed with the GLOOME program v1.266^94^ using the implemented parsimony method.

Most importantly, given the level of sharing among the eight focal species, the total number of expressed miRNAs identified in our dataset were grouped into different categories for downstream analyses. A miRNA was defined as “conserved” when found in all the eight species, otherwise, we referred to it as a “non-conserved” miRNA. A miRNA identified in only one species is considered as being “species-specific”.

Since many miRNA genes cluster together in the genome and are transcribed as polycistrons^95^, we also identified miRNA gene clusters by grouped miRNA genes on the same strand within 50 kb of each other. Usually, the distance between two adjacent miRNA genes is shorter than 5 kb or longer than 50 kb^21^. We compared the clustering performance of “conserved” and “non-conserved” miRNA genes, as only a few “species-specific” miRNA genes are clustered.

### Transcriptome assembly

The transcriptomes of each species were assembled using a reference-based approach by applying the species’ corresponding genome when available. Because the reference genomes of *A. alluaudi* and *A. siquia* are unavailable, we used the genome of *O. niloticus* as the reference genome for *A. alluaudi* and the genome of *A. citrinellus* as the reference genome for *A. siquia*. First, raw RNA-Seq reads were processed by trimming the adaptors and low-quality bases using the program Trimmomatic v0.36^94^ with the parameter “LEADING:3 TRAILING:3 SLIDINGWINDOW:4:15 MINLEN:36”. Second, filtered reads were mapped to the reference genome using Hisat v2.1.0^96^. The alignment files were processed by StringTie v1.3.3b^97^ in order to construct the gene models and thus infer the transcriptomes.

### Quantification of miRNAs and mRNAs expression

For each individual, the expression of each transcript included in the previously constructed gene model was quantified using StringTie. To account for both gene length and the per sample differences in depth of coverage, the obtained RNA-Seq raw expression values were normalized using the transcripts per million (TPM) approach. The expression of miRNAs was quantified and normalized for each individual using the *quantifier.pl* script in miRDeep2 package. For *A. alluaudi* and *A. siquia*, for which we used different reference genomes, the highest normalized expression value was chosen for each mature miRNA.

### Retrieval of sequences and phylogenetic reconstruction

The sequences of each transcript were extracted from the StringTie assemblies using the *gffread* module of the Cufflinks v2.2.1 package, except for *A. alluaudi* and *A. siquia*. In these two species, we applied a *de novo* approach to assemble the transcriptomes using Trinity v2.6.4^98^, thus obtaining the exact sequences for each species. For each species, all transcripts were aligned to peptides of *O. niloticus* using Blastx^99^. The coding sequences (CDS) from *O. niloticus* were aligned to the cichlid transcripts setting an e-value cutoff of 1e^−10^. The pairs with a reciprocal best hits (RBH) were identified as one-to-one orthologs to *O. niloticus* transcripts. For each orthologous transcript, the CDS was recognized according to the tBlast result, and the sequences downstream of this region were regarded as 3′ UTR.

We used coding sequences of orthologs present in all eight species to reconstruct their phylogeny. The sequences were aligned using Mafft v7.310^100^ and the alignments were concatenated. We reconstructed the maximum likelihood tree of the eight focal species using the program RAxML v8.2.4^101^. The four-fold degenerate sites were extracted from the alignments to estimate the relative divergences times using the MCMCtree program from the PAML v4.9 package^102^.

### Target prediction of miRNAs

We aligned the 3′ UTR sequences of each orthologous gene using Mafft. Then, we used mature miRNA sequences and multiple sequence alignment of 3′ UTR to predict miRNA target sites using TargetScan 6^103^. In principle, the seed sequences should be perfectly matched for the target prediction. The potential functional pairs of miRNA-mRNA were identified according to the target prediction.

### Correlation between miRNA-mRNA expression

We estimated the expression relationships between functional miRNA-mRNA pairs found in all eight cichlid species. For each miRNA-mRNA pair, Pearson’s correlation coefficient of miRNA and mRNA expression was calculated among all individuals. Also, the correlation coefficients of functional miRNA-mRNA pairs across species were estimated after calculating phylogenetically independent contrasts using the “pic” function in the R-package ape^104^.

### GO enrichment analysis

For each transcript, we estimated the emergence rate of species-specific miRNA targets by calculating the number of species-specific targets divided by the total length of 3′ UTRs. Enrichment analysis was performed on the top 5% genes showing the highest emergence rate of species-specific miRNA targets. We identified significantly over-represented gene ontology (GO) terms in the identified genes (test set) when compared to the whole gene set (baseline set). To assess significance, we used the Fisher’s exact test implemented in g:Profiler^105^. As a baseline reference set, we used the Nile tilapia.

## Supplementary information

Supplementary Fig. S1–S5

Supplementary Tables S1–S5

## Data Availability

Raw Illumina sequences have been deposited into the NCBI’s Sequence Read Archive (SRA) database with accession numbers PRJNA559612 (RNA-Seq) and PRJNA559778 (miRNA-Seq). The transcriptome assemblies and the normalized expression values of both miRNA and mRNA samples have been uploaded to the Dryad Data Repository (10.5061/dryad.qc23720).
