# Supplementary material for: Conservation and novelty in the microRNA genomic landscape of hyperdiverse cichlid fishes
Source: Sci Rep. 2019 Sep 25;9:13848. doi: 10.1038/s41598-019-50124-0 (PMC6761260; doi:10.1038/s41598-019-50124-0)
Supplement: Supplementary file 1 — Supplementary Fig. S1–S5 [file 41598_2019_50124_MOESM1_ESM.pdf]

Supplementary figures for the article:

Conservation and novelty in the microRNA genomic landscape of hyperdiverse cichlid fishes

Peiwen Xiong[1], Ralf F. Schneider[1,2], C. Darrin Hulsey[1], Axel Meyer[1], Paolo Franchini[1*]

[1]Chair in Zoology and Evolutionary Biology, Department of Biology, University of Konstanz, 78457 Konstanz, Germany

[2]current address: GEOMAR Helmholtz Centre for Ocean Research Kiel, Marine Ecology, Germany

[*]Correspondence: paolo.franchini@uni-konstanz.de

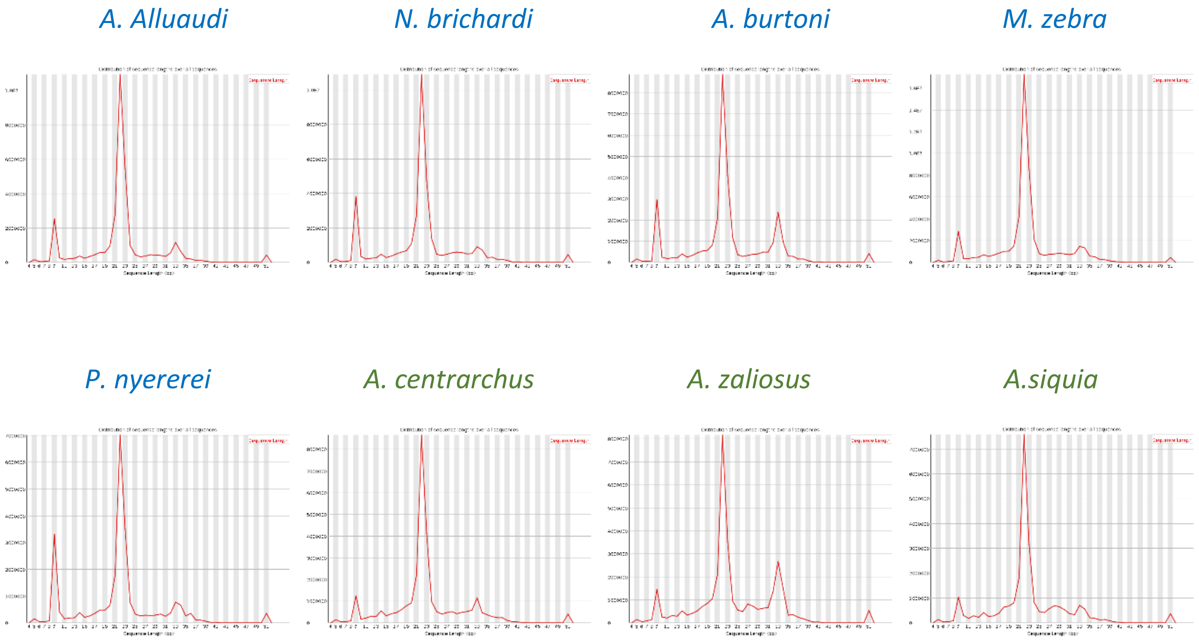

Fig. S1. Length distribution of sequencing reads of small RNAs in eight cichlid species (African and Neotrpical species are labeled in blue and green, respectively).

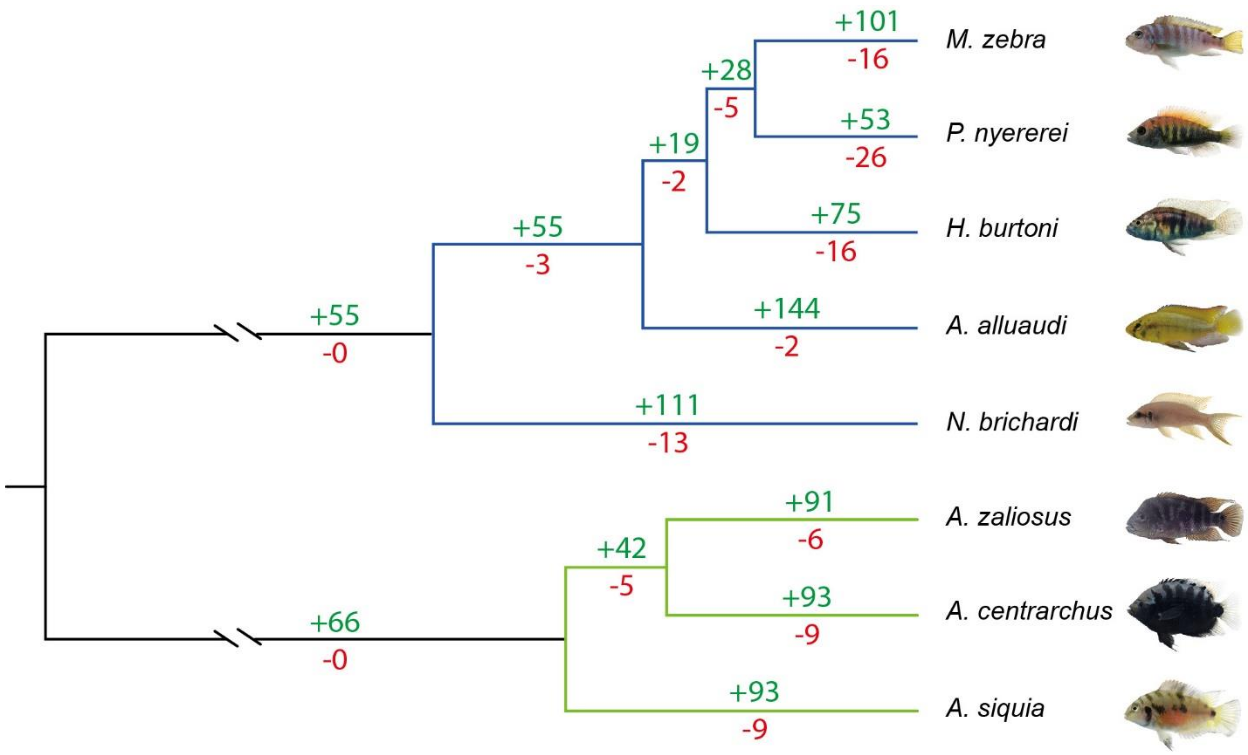

Fig. S2. Parsimony inference of miRNA gain and loss events across the phylogeny. For each branch, green and red numbers indicate gain and loss of miRNAs, respectively.

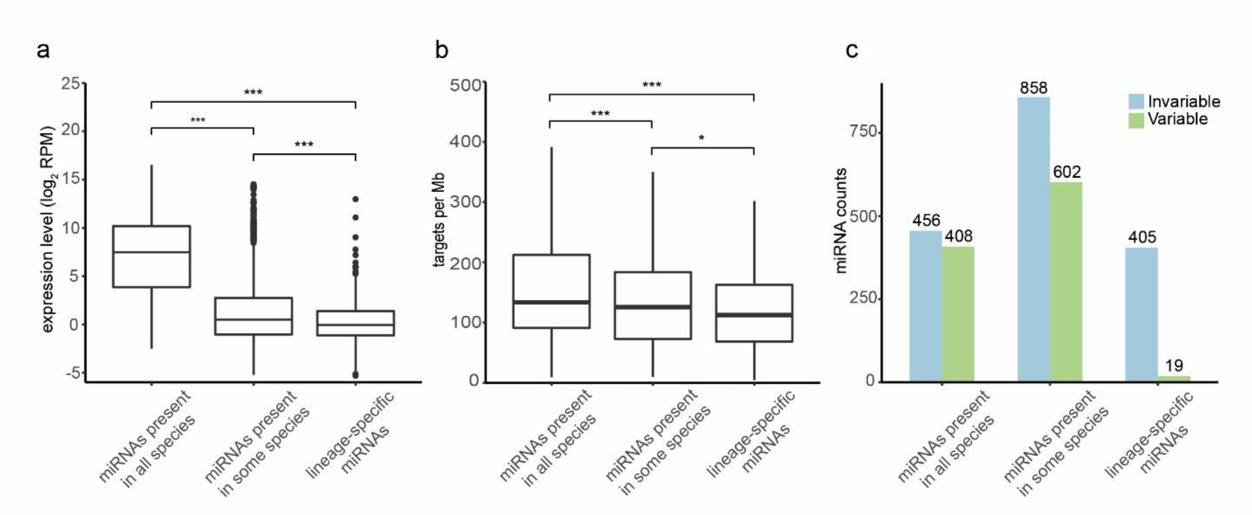

Fig S3. Differentiation in miRNAs characteristics. Similar to figure 3, the miRNA expression level, density of miRNA target sites, and mature miRNA sequence variability were compared among different groups of miRNAs, including the group of miRNAs present in some species (2-7 species).

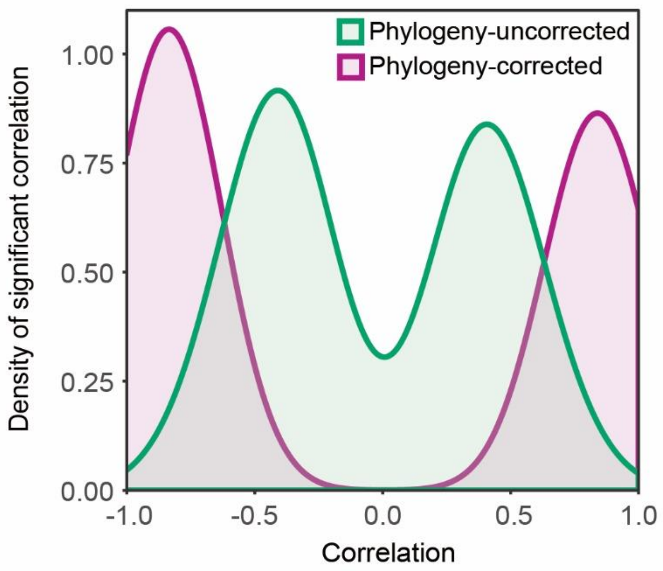

Fig. S4. Correlation between mRNA and miRNA expression. The density (width=0.1) of significant Pearson correlation coefficients ($p < 0.05$) between miRNA and mRNA expression for all the miRNA-mRNA targeted pairs conserved in the eight cichlids. The correlations were estimated in two ways: using the expression of all individual (n=48) (green), and using a phylogeny-based correction (purple) that only examined species means (n=8). For both estimations, the distribution is skewed to having a greater number of negative correlations which is what one would expect.

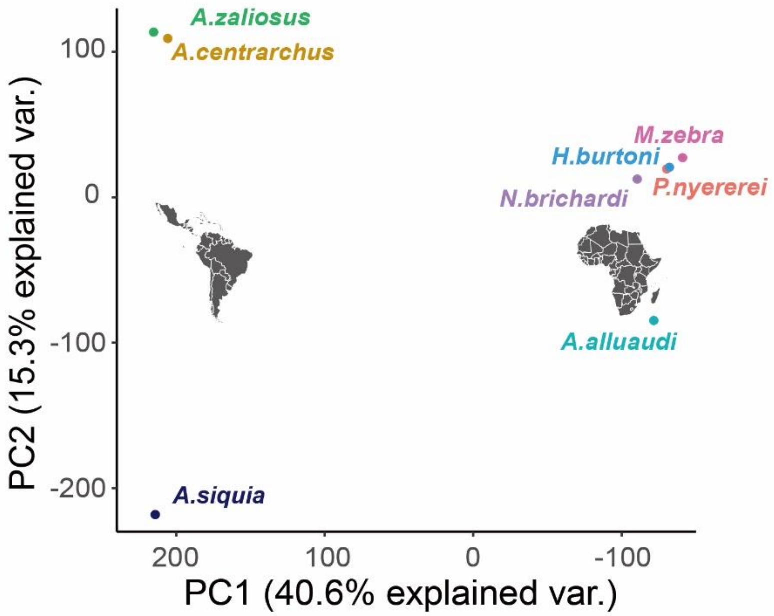

Fig. S5. PCA of miRNA-mRNA target pattern between 108 miRNAs and 2,262 mRNAs that were present in all eight studied cichlid species.
